# Chitosan as a Protective Matrix for the Squaraine Dye

**DOI:** 10.3390/ma14051171

**Published:** 2021-03-02

**Authors:** Halina Kaczmarek, Patryk Rybczyński, Piotr Maćczak, Aleksander Smolarkiewicz-Wyczachowski, Marta Ziegler-Borowska

**Affiliations:** 1 Faculty of Chemistry, Nicolaus Copernicus University in Toruń, Gagarina 7, 87-100 Toruń, Poland; pat_ryb@doktorant.umk.pl (P.R.); pmacczak@doktorant.umk.pl (P.M.); 291065@stud.umk.pl (A.S.-W.); martaz@umk.pl (M.Z.-B.); 2 Water Supply and Sewage Enterprise LLC, Przemysłowa 4, 99-300 Kutno, Poland

**Keywords:** chitosan, squaraine dye, fluorescence, morphology, photochemical stability, thermal stability, dye release

## Abstract

Chitosan was used as a protective matrix for the photosensitive dye-squaraine (2,4-bis[4-(dimethylamino)phenyl]cyclobutane-1,3-diol). The physicochemical properties of the obtained systems, both in solution and in a solid-state, were investigated. However, it was found that diluted chitosan solutions with a few percent additions of dye show an intense fluorescence, which is suppressed in the solid-state. This is related to the morphology of the heterogeneous modified chitosan films. The important advantage of using a biopolymer matrix is the prevention of dye degradation under the influence of high energy ultraviolet (UV) radiation while the dye presence improves the chitosan heat resistance. It is caused by mutual interactions between macromolecules and dye. Owing to the protective action of chitosan, the dye release in liquid medium is limited. Chitosan solutions with a few percent additions of squaraine can be used in biomedical imaging thanks to the ability to emit light, while chitosan films can be protective coatings resistant to high temperatures and UV radiation.

## 1. Introduction

Chitosan (CS) is an example of a biopolymer that arouses great interest among scientists and producers for at least two decades due to its unique properties such as biodegradability, biocompatibility, and nontoxicity—which means it is safe for humans—antibacterial, and antifungal properties. CS is highly available from biological renewable sources, thus its production cost is low, and can be easily modified owing to the presence of functional groups in chemical structure. This is evidenced by a huge amount of reviews and monographs in recent years, which describe the possibility of new applications in many areas of the industry, science, and medicine [1,2,3,4,5,6,7,8,9,10]. Therefore, it is undoubtedly an environmentally friendly material that meets the requirements of green chemistry.

In addition to the well-known applications for the production of a wound dressing, cell growth scaffolds, controlled-release drug carriers, hygiene products, or biodegradable packaging [1,2,3,5,6,7], there are original proposals for using this polysaccharide as flocculants in the treatment of drinking water [11,12], three dimensional-printed chitosan hydrogels for development of tissue engineering [13], as a valuable material for dentistry [14], targeted protein and gene delivery [15], or as photosensitizer carrier for Photodynamic Therapy (PDT) [16], material for refining fibers and fabrics (production of intelligent clothing) [17], in veterinary (e.g., in fish farming) [18], and as plant protection products [19]. Chitosan can be used in various physical forms (films, granules, microcapsules, gels, and aerogels), alone or in complex multi-component systems (as a matrix of other active ingredients, polymer blends, composites, and nanocomposites) [17,20,21].

Recently, luminescent chitosan is of great interest because it can be used as a fluorescent probe in the imaging of biological objects or metal ion sensors [22,23,24,25,26]. Chitosan fluorescence is the result of its chemical modification by photosensitive organic compounds, for example, phenothiazine, fluorene, fluorescein, rhodamine, and their derivatives [23,26,27,28,29]. It has also been reported that chitosan alone (not dye-labeled) in the form of small micelles obtained in long-term heat treatment shows fluorescence [24].

In this work, we used another photosensitive compound—a selected squaraine dye—which was incorporated into the chitosan matrix or solution. Squaraine dyes are derivative of square acid (3,4-dihydroksycyclobutene-1,2-dion), containing a four-membered ring with two oxygen heteroatoms in the center of the molecule with two substituted aromatic rings. They are characterized by absorption and intensive fluorescence in the visible range, thanks to which they find various applications, e.g., as photovoltaic materials (solar cells), molecular sensors, or probes (labels) in biomedical imaging [30,31,32,33,34]. The big advantage of squaraine dyes is their non-toxicity in the dark but the ability to generate reactive oxygen species (including free radicals) under the influence of light contributes to the death of pathogenic cells, hence, the possibility of their use in medicine, particularly in PDT, which is a non-invasive anti-cancer method [34].

This work aimed to obtain chitosan modified with fluorescent squaraine dye: 2,4-bis[4-(dimethylamino)phenyl]cyclobutane-1,3-diol (DMASQ) and to characterize the physicochemical properties of the prepared system in solutions and a solid-state (in the form of thin films), mainly photochemical and thermal stability. Moreover, the morphology of CS + DMASQ films and the kinetic of dye release from the biopolymer matrix were investigated. Our approach is based on a simple method of physical modification, contrary to the more complicated chemical functionalization of this polysaccharide of great practical importance.

## 2. Materials and Methods 

### 2.1. Materials 

Chitosan of low molecular weight (MW = 50 kDa) and deacetylation degree of 75–85% was purchased from Sigma-Aldrich (Saint Louis, MO, USA). 3,4-Dihydroxy-3-cyclobutene-1,2-dione (squaric acid), *N,N*-dimethylaniline, 1,3-diphenylisobenzofuran (DPBF) were supplied by Sigma-Aldrich Solvents: Acetic acid, n-butanol, benzene, hexane, dichloromethane, chloroform, methanol, tetrahydrofuran, dimethyl sulfoxide (DMSO), high purity grade, purchased from POCH™ (Avantor™ Performance Materials, Gliwice, Poland) and used without any additional purification.

### 2.2. Synthesis of Symmetric Squaraine Dye: 2,4-Bis[4-(dimethylamino)phenyl]cyclobutane-1,3-diol (DMASQ)

**Figure 1:**
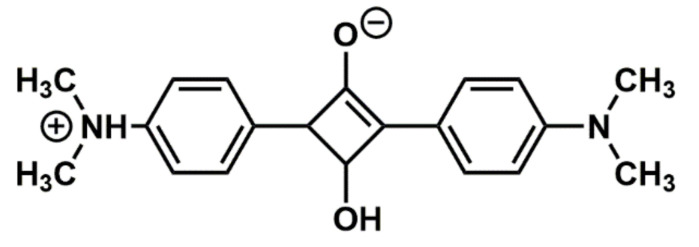
Chemical structure of 2,4-bis[4-(dimethylamin)phenyl]cyclobutane-1,3-diol (DMASQ).

The squaraine dye (Figure 1) was obtained in the condensation reaction according to the method described by Sprenger and Ziegenbein [35]. The modification of the method involves extending the synthesis time, which allowed for greater reaction efficiency.

Squaric acid (11.5 mg, 0.10 mmol) and *N,N*-dimethylaniline (29 mg, 0.24 mmol) were heated in a mixture of n-butanol with benzene (in a volume ratio of 1:1) at 120 °C for 24 h with Dean-Stark trap (Boreal Science, St. Catharines, ON, Canada). The resulting deep blue product was filtered and purified by recrystallization from methylene dichloride and hexane (4:1 *v*/*v*). The crystalline blue product (DMASQ) was dried in the absence of light. DMASQ was obtained with 56% yield and melting point 294–296 °C (ref. [30] T_m_ = 276 °C). The structure of DMASQ was confirmed by proton nuclear magnetic resonance analysis: ^1^H-NMR, DMSO_d-6_, (δ, ppm): 3.25 (d, 12H, 4 × CH_3_); 6.78 (d, J = 9.1 Hz, 4H, CH_Ar_); 8.40 (d, J = 9.1 Hz, 4H, CH_Ar_).

### 2.3. Preparation of Chitosan Films

A 1% stock solution of chitosan in 1% acetic acid was prepared by mechanical stirring for 24 h at room temperature and then filtered. The filtrate was used to prepare films by pouring onto leveled Petri dishes. Separately, a solution of the dye in methanol was prepared by stirring for 30 min. The solutions of CS and DMASQ in the various proportions were mixed and cast for solvent evaporation. Modified chitosan films contained 1, 2, 5, and 10 wt.% of dye.

All obtained solid films were soaked in 1 M sodium hydroxide solution for 4 h, then washed with distilled water until neutral and dried at 60° C. The pure chitosan film was colorless and transparent while the dye-modified chitosan was blue. The thickness of the obtained films was about 1–1.5 μm. To compare the properties of samples of different compositions, films of equal thickness were selected. The films obtained in this way were characterized by FTIR, Raman, UV-Vis spectroscopy, and spectrofluorimetry. The morphology was examined by scanning electron microscopy (SEM) and atomic force microscopy (AFM). The materials were subjected to polychromatic radiation and thermogravimetric analysis to determine the photochemical and thermal stability, respectively.

### 2.4. Spectroscopic Analysis

Attenuated Total Reflection Infrared spectra of solid films (chitosan and dye-doped chitosan) were obtained by Perkin Elmer Spectrum Two^®^ spectrophotometer (Perkin Elmer, MA, USA) in the range of 400–4000 cm^−1^ with a resolution of 4 cm^−1^. Each spectrum was obtained by collecting 64 scans. Correction of the baseline, normalization, and determination of the integral intensity of selected absorption bands was performed with the software provided by the manufacturer.

Raman spectra of studied films were recorded using Raman spectrometer Senterra by Bruker Optik (Bruker, Billerica, MA, USA) with the laser operating at 785 nm in the range of 90–3500 cm^−1^ range. Laser power was 100 mW, co-additions: 10, integration time: 5 s. The spectrometer was equipped with a thermoelectric cooled CCD (charge-coupled device) detector and a video camera that allows us to observe and choose the place of measurement.

UV-Vis spectra of samples in solution were recorded with a Shimadzu UV-1601PC spectrophotometer (Shimadzu, Kioto, Japan). The dye concentration in the solutions was in the order of 10^−4^ M. Standard 1 cm thick quartz cuvettes were used.

Diffuse reflectance UV-Vis spectroscopy (DRS) of solid films was performed using V-750 UV–visible Spectrophotometer, (JASCO, Tokyo, Japan) with ISV-922 Integrating Sphere coated with barium sulfate (JASCO, Tokyo, Japan).

^1^H-NMR spectroscopy of DMASQ in deuterated DMSO (d-6) solution was done with Bruker Avance III 700 MHz (Bruker, Billerica, MA, USA).

### 2.5. Morphology Characterization

SEM images at different magnifications were taken with a scanning electron microscope produced by LEO Electron Microscopy Ltd., (Cambridge, UK), model 1430 VP operating at low pressure and an accelerating voltage of 10–20 kV. The samples without sputtering were tested.

Atomic Force Microscopy, AFM (MultiMode, Nnaoscope IIIa Veeco Metrology Inc., Santa Barbara, CA, USA) was used to visualize the surface morphology of the sample and to determine the roughness parameters (nm): Arithmetic mean—R_a_, root mean square—R_q,_ and maximum peak height—R_max_ which is the highest peak above the mean line in the profile. The scan area was 2 × 2 μm^2^. 

From the set of SEM and AFM photos taken, the most characteristic and repeatable images of the sample surface were selected for presentation.

### 2.6. Fluorescence

Fluorescence spectra in solutions (with a concentration of 6 × 10^−4^ M) and in the solid-state were recorded on an F-7000 FL spectrophotometer (Tokyo, Japan) in the wavelength range of 220–550 nm. The excitation wavelength was 480 and 600 nm.

### 2.7. Photochemical Stability 

To estimate the photochemical stability of the samples, a high-pressure mercury vapor lamp HPK 125 W (Philips, Amsterdam, The Netherlands) was used. This lamp emits high-energy polychromatic UV radiation in the range of 248–578 nm. The incident radiation intensity, measured with HD 9021 radiometer (Delta, Wevelgem, Belgium), was 30, 20, and 5 W/m^2^ for UVA, UVB, and UVC, respectively. Samples were exposed in the air atmosphere. After each exposure time, the IR and UV-Vis absorption spectra were measured.

To quantify the effect of radiation on the dye, the percentage degradation efficiency (DE) was determined as:

(1) $$\mathrm{DE}\;=\;\frac{A_{0}-A_{t}}{A_{0}}\times 100\%$$

where A_0_ is the absorbance at the maximum wavelength (640 nm) for the unirradiated sample and A_t_—after t irradiation time.

### 2.8. Thermal Analysis

Thermogravimetric analysis of CS films was performed using a TA Instruments type SDT 2960 Simultaneous TGA-DTA (New Castle, UK) in the air atmosphere. The heating rate was 10 °C/min, temperature range 20–800 °C, sample mass about 5 mg. Thermogravimetric (TG), differential thermal analysis (DTG), and temperature difference (DTA) curves were recorded simultaneously. 

### 2.9. Release of DMASQ from the Chitosan Matrix

During the tests, always the same weight (0.03 g) of the polymer film with a different content (1–10%) of the DMASQ dye was weighed. Each sample was placed in a quartz cuvette and 3.5 cm^3^ of methanol was added. The release was studied at room temperature by UV-Vis spectroscopy. The spectra were recorded at 5 min intervals. The absorption at 640 nm, i.e., at wavelength characteristic for the dye released from the chitosan matrix, was determined from the spectra. The dye concentration has been determined on the base of the calibration curve and calculated molar absorption coefficient (ε). ε of DMASQ at 640 nm in methanol was 75,746 M^−1^·cm^−1^.

## 3. Results

### 3.1. General Comments on the Properties of DMASQ Dye

Obtained DMASQ was a deep blue crystalline powder, insoluble in water but soluble in organic solvents among others in chloroform, tetrahydrofuran, ethanol, and methanol. Introduced into chitosan, it caused the blue color both in the solution and in the solid film. The spectroscopic properties of DMASQ result from its chemical structure—the molecule, in addition to the central squaric ring (cyclobutene), contains two aromatic rings substituted with dimethylamine groups in the para position, which makes the molecule symmetrical, coplanar, and apolar (Figure 1). Due to the presence of π electrons in rings and non-bonding electron pairs in amino groups, the molecules occur in several resonance structures [36]. A cyclobutane ring with an electron acceptor nature is the part of the molecule with the highest electron density, while the terminal dimethylammonium phenyl groups have a donor character and partially positive charge localized on nitrogen atoms. This enables different interactions with molecules in the adjacent surrounding.

The chemical structure of DMASQ dye was characterized by ^1^H-NMR and Attenuated Total Reflectance-FTIR (ATR-FTIR) spectroscopy. ^1^H-NMR spectra in DMSO exhibits the following signals (δ, ppm): 3.25 (d, 12H, 4 × CH_3_); 6.78 (d, J = 9.1 Hz, 4H, CH_Ar_); 8.40 (d, J = 9.1 Hz, 4H, CH_Ar_) which confirm the structure shown in Figure 1. Infrared spectroscopic studies also confirm the structure of the dye obtained. ATR-FTIR spectrum of DMASQ shows intensive bands in 500–2000 cm^−1^ range, in particular: 1800–1600 cm^−1^ corresponding to C=O stretching vibrations, 1500–1600 cm^−1^—overlapping vibrations of phenyl rings, and unsaturated C=C bonds. The strong absorptions at 1593 cm*^−^*^1^ and 1529 cm*^−^*^1^ are attributed to the mixed vibrations of the squarate ring. A band in the range of 800–860 cm^−1^ is typical for p-substituted aromatic rings. The tertiary amines exhibit bands in the fingerprint region (700–1300 cm^−1^), thus absorption at 1180–1360 cm^−1^ corresponds to the C–N stretching vibration overlapping with C–O vibrations. There are no absorption bands in the area of the hydroxyl groups (3000–3500 cm^−1^), which additionally indicates no adsorbed moisture in the dye sample. Also, tertiary amines do not absorb in the range above 3200 cm^−1^. These results are in line with literature data for another derivative of square acid [37,38].

### 3.2. Spectroscopic Characteristic of DMASQ Dye in Solutions

DMASQ in diluted solutions shows intense absorption in the visible range, which is related to the π→π* transition (HOMO→LUMO) of the carbonyl and phenyl groups [39]. The main absorption band in the UV-Vis range appears at 627 nm (in tetrahydrofuran, THF), 630 nm (in chloroform), and 631 nm (in ethanol) indicating the solvatochromic effect, which has been described in detail in previous work [36]. The occurring absorption corresponds to the so-called therapeutic window (600–850 nm) required in photosensitizers intended for photodynamic therapy. Less important from the point of view of biomedical applications is absorption in the shortwave range (200–300 nm), where also chitosan absorbs. As these spectra have been previously discussed and supported by theoretical calculations [36], there is no need to quote them here. Moreover, typical organic solvents are excluded because they do not dissolve CS.

**Figure 2:**
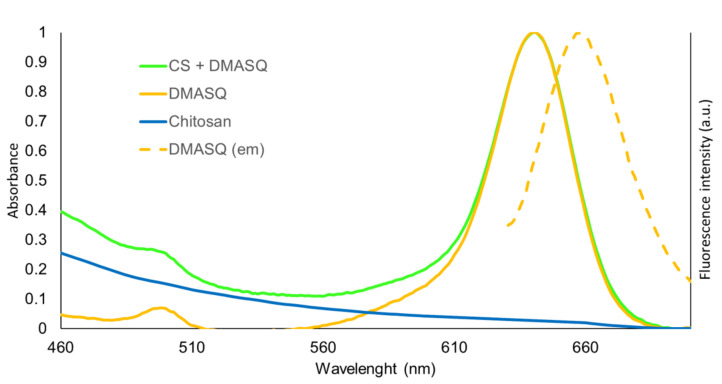
Normalized absorption spectra (solid lines) of DMASQ, chitosan, and chitosan (CS) + DMASQ in the solution of acetic acid + methanol (1:1) and emission spectrum of DMASQ under excitation wavelength 600 nm (dashed line). DMASQ concentration: 6 × 10^−4^ mol/dm^3^, the concentration of acetic acid used—1%.

Below we present an absorption spectrum of diluted solutions of DMASQ in the solvent with the same composition as that used for the modified chitosan, i.e., a mixture of acetic acid and methanol at a 1:1 volume ratio (Figure 2). For comparison, the spectrum of CS itself is also provided. Pure chitosan practically does not absorb above 400 nm, while DMASQ exhibits a strong band with maximum absorption at 640 nm, which also appears in the spectrum of the solution containing CS + DMASQ. This absorption band does not change its position and shape because the same solution mixture was used. It means that interactions of solute with solvent molecules are stronger than between CS and DMASQ in such diluted solutions.

The most important feature of the DMASQ dye is its ability to emit light. The fluorescence in solutions also depends on the type of solvent used because the Stokes shifts depend on the chemical micro circumstances. Although in symmetric molecules of dye the polarity is very low, the presence of polarized bonds (C–O, N–C) causes local polarity changes. In these studies of fluorescence, the same solvent composition as in the absorption studies (i.e., acetic acid + methanol 1:1) was used. The emission spectra of CS + DMASQ were obtained using 480 and 600 nm excitation wavelengths (Figure 2 shows the fluorescence at 600 nm excitation). The solution of chitosan alone does not fluoresce while the CS doped with DMASQ exhibits the same emission band as squaraine dye with the wavelength at the absorption maximum: 510 nm at λ_ex_ = 480 nm or 660 nm at λ_ex_ = 600 nm. The emission spectrum of DMASQ, shown in Figure 2, after normalization exactly matches the spectra of the dye-doped CS. 

These observations are consistent with Huang’s statement who described that chitosan is too weakly fluorescent for its emitting properties to be of practical importance [24]. 

### 3.3. Characteristic of Chitosan-DMASQ Films 

#### 3.3.1. Surface Morphology

**Figure 3:**
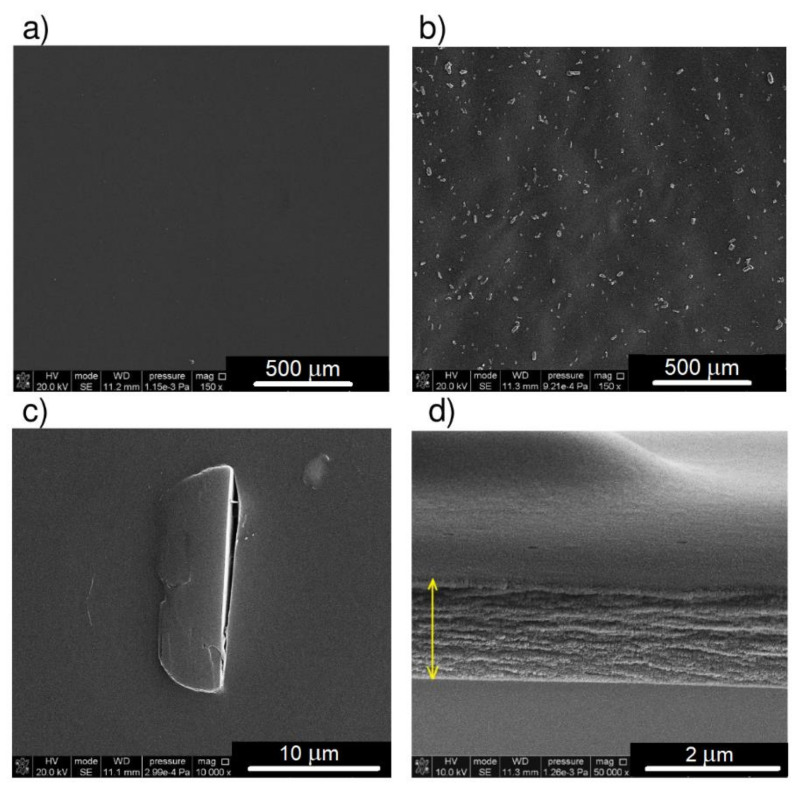
SEM images of chitosan (**a**) and chitosan with 5% DMASQ at 150× magnification (**b**); single DMASQ crystal embedded in CS film, magnification 10,000× (**c**) and cross-section of CS + 2% DMASQ film, magnification 50,000×, the arrow shows the thickness of the film (**d**).

**Figure 4:**
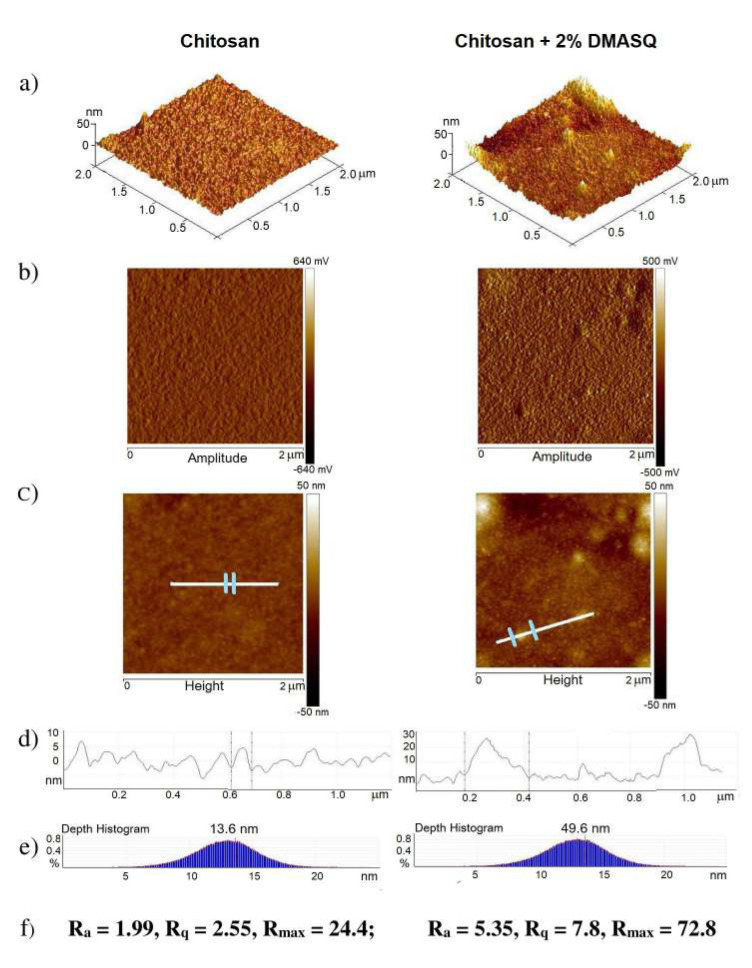
Semi-3D atomic force microscopy (AFM) (**a**), amplitude (**b**), topography (**c**) images; cross-sections (**d**) taken along the lines marked on the height image shown in Figure 6c; depth histograms (**e**) and roughness parameters in nm (**f**) of chitosan (left column) and DMSAQ doped chitosan (right side).

Chitosan alone does not show any special details of the microstructure—the films are flat and smooth without surface defects, which is shown by both SEM and AFM images (Figure 3 and Figure 4). In the case of the films with the addition of a dye, a bluish color is observed even with the naked eye, with an intensity depending on the amount of squaraine introduced. SEM images of CS with the dye allowing us to observe the heterogeneity of the system (Figure 3b). Despite the homogeneity of the mixture in the solution, after pouring out and gradual evaporation of the solvent, the DMASQ particles precipitate and form more or less regular microcrystals the shape of which is visible in images at high magnification (Figure 3c). The estimated size of the dispersed DMASQ crystals in the CS film is in the range of 4–20 μm and does not depend on the ratio of the components. The cross-sections of the chitosan films (Figure 3d) show a relatively homogeneous internal structure and simultaneously allow for a precise thickness measurement (approximately 1.5 μm).

AFM images were obtained for different scan areas but for the presentation of the most representative details of surface morphology, the results for the scan area of 4 μm^2^ are shown (Figure 4). As can be seen, unmodified CS shows a slight surface roughness, as evidenced by the AFM images and the determined roughness parameter values (R_a_ = 1.99 nm and R_q_ = 2.55 nm). Typical elevations and depressions on the surface are about 5–10 nm (Figure 4d). The depth histogram allows us to observe the distribution of the data points on the specimen surface (Figure 4e). On the ordinate axis in this figure is the percentage of all points of given deviation from a flat surface while on the abscissa axis is a depth in nm. The maximum on this curve indicates that most deviations have a medium depth value of 13.6 nm. The highest hill (R_max_) in the observed area is just over 20 nm.

Doped chitosan film exhibits greater surface imperfections associated with embedded DMASQ particles. The roughness parameters increase approximately 3 times compared to pure chitosan.

#### 3.3.2. FTIR, Raman and UV-Vis Spectroscopy

**Figure 5:**
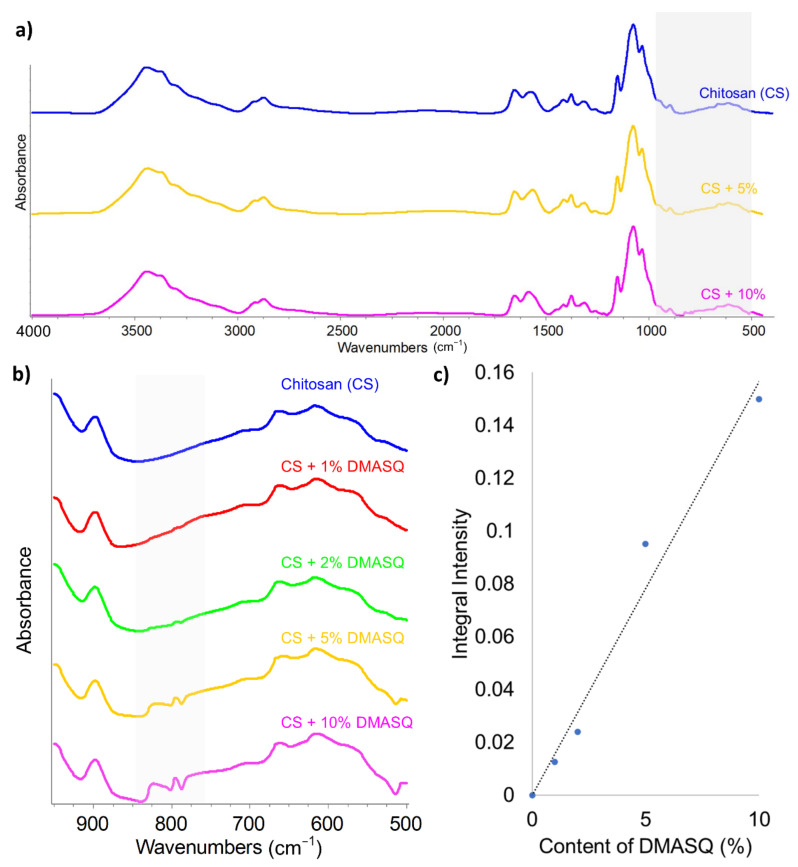
FTIR spectra of CS and CS with different content of DMASQ in 4000–400 cm^−1^; (**a**) and 500–1000 cm^−1^ range—fragment shaded in the Figure 5a (**b**); dependence of integral intensity of band at 795 cm^−1^ vs. DMASQ content in CS (**c**).

FTIR spectrum of chitosan films exhibits the typical bands characteristic for polysaccharides: Strong band in the range of 3100–3600 cm^−1^ (corresponding to O–H stretching vibrations), 3309 cm^−1^ (N-H_str_), 2875 cm^−1^ (CH_2_ in pyranose rings), 1650 cm^−1^ (C=O in amide), 1574 cm^−1^ (NH_2str_), 1556 (NH_def_), 1375 cm^−1^ (CH_3def_), and 1150–1027 cm^−1^ (glycoside C–O–C_def_). The introduction of DMASQ to the chitosan matrix causes negligible modification of the FTIR spectrum because the dye absorption bands are obscured by those of chitosan (Figure 5a,b). Only at the higher dye content (5–10%), an arm appears at 1693 on the peak with a maximum at 1650 from CS. The changes in the range of 950–500 cm^−1^, where absorption band characteristic for DMASQ is very strong, are also visible. In CS film doped with dye, an absorption band appears at 795 cm^−1^, the intensity of which increases with increasing dye content. The dependence of the peak area on the amount of squaraine, presented in Figure 5c, indicates compliance with the Beer-Lambert law.

**Figure 6:**
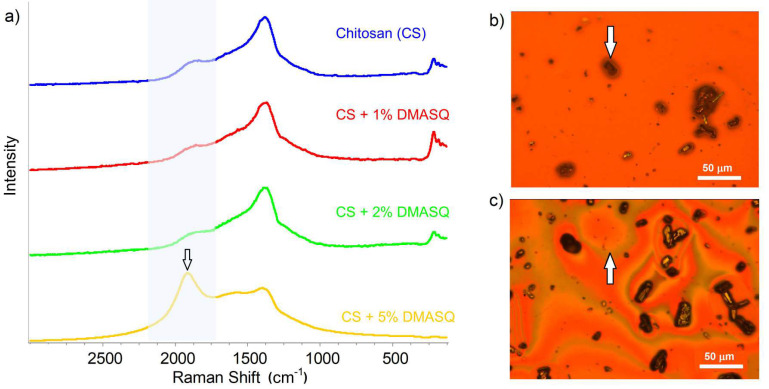
Raman spectra (**a**) of CS and CS containing 1–5% DMASQ films in 2500–500 cm^−1^ range (the shaded part indicates a band with a maximum at 1910 cm^−1^ from DMASQ); microscopic image of CS + 2% DMASQ (**b**) and CS + 5% DMASQ (**c**) where the arrow points to the interphase region.

Complementary information is provided by Raman spectroscopy (Figure 6). The strong absorption band in this region at the wavenumber of 1370 cm^−1^ comes from chitosan. This is consistent with the reports of other authors [37,40,41]. The band with the maximum at 1910 cm^−1^ (shaded), characteristic for the DMASQ dye, appears in the spectra of doped CS films. A clear peak is observed only at the dye content from 5% upwards. These spectra confirm that DMASQ dye has been successfully incorporated into CS bulk. Owing to combining the Raman spectrometer with a microscope, it was possible to select the appropriate place of the sample to collect the spectrum. Simultaneously microscopic observation showed the existence of interfacial areas in addition to visible crystals. This region is small and seen as a thin coating on a crystal in the case of 2% of the dye (Figure 6b) and much wider but fuzzy in the system with 5% DMASQ (Figure 6c). The Raman spectrum measured in this area (e.g., at the point indicated by an arrow in a microscope photo, Figure 6b,c) contains the intense 1910 cm^−1^ band.

**Figure 7:**
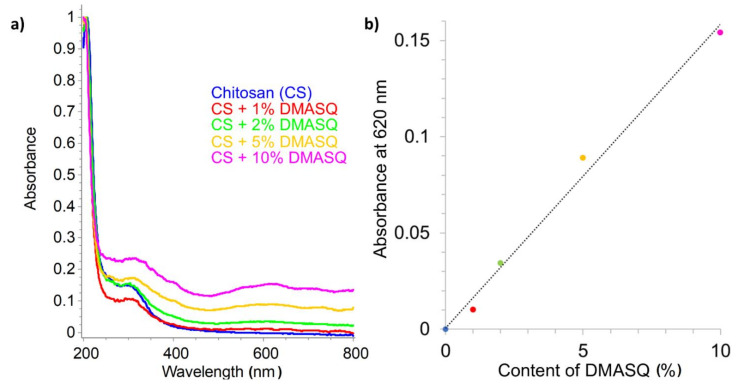
Diffuse reflectance spectra of chitosan films containing 1–10% DMASQ normalized at 210 nm (**a**) and the dependence of absorbance at 620 nm on the percentage of dye in the CS matrix (**b**).

Moreover, UV-Vis absorption spectra of all CS films were monitored using diffuse reflectance (DR) spectroscopy (Figure 7a), which is based on the measurement of the radiation scattered by the surface of a solid sample and reflected from BaSO_4_ standard. As can be seen, with the increase of the dye content in the CS, the turbidity of the sample increases (observed as a background rise), which is caused by the heterogeneity of the system. At the same time, a wide but low intensive band with a maximum of 620 nm appears. It shows a linear dependence on the dye content in the sample (Figure 7b). The band at approximately 300 nm, visible in the spectra of all samples, is due to the presence of remaining acetyl groups from chitosan. 

Unfortunately, practically no fluorescence was observed for the obtained solid CS films with the addition of DMASQ, contrary to solutions of this blend. This is because the dye crystallizes in the polymer matrix when evaporating the solvent, which causes sample heterogeneity and the scattering of incident radiation, competing with absorption. Moreover, in the condensed phase, the excited molecules mutually quench each other or lose their energy in the internal conversion processes, which results in a lack of radiation emission, in contrast, to dilute solutions.

### 3.4. Photochemical Stability 

**Figure 8:**
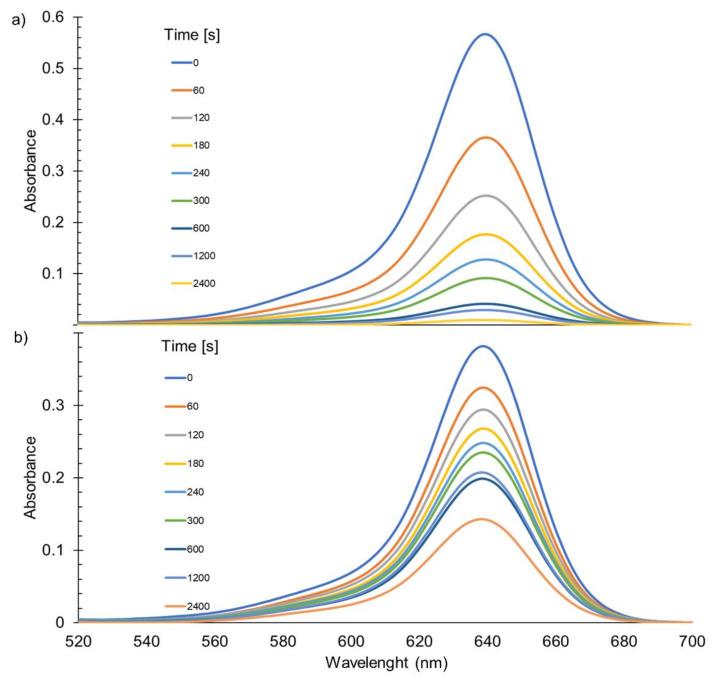
Changes in UV-Vis absorption spectra of DMASQ (**a**) and CS + 2% DMASQ (**b**) solutions (in acetic acid/methanol 1:1 v/v) during UV-exposure.

**Figure 9:**
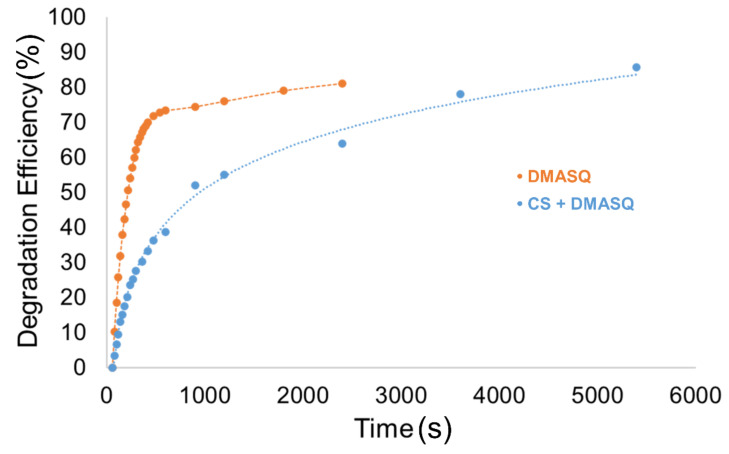
Kinetics of dye photodegradation in solution with and without chitosan as a function of UV-irradiation time (degradation efficiency was calculated according to formula 2).

To determine the photostability of the tested systems, they were exposed to high-energy UV radiation, which can be used to sterilize various medical accessories and products. Both films and CS solutions were exposed, and the course of photochemical reactions was monitored by FTIR and UV-Vis spectroscopy, respectively. Figure 8a,b shows the decrease of the absorption band at 640 nm in a solution of DMASQ and DMASQ doped CS caused by the photobleaching effect. The changes of absorbance at band maximum were used to calculate the percentage degree of dye decomposition, which is presented in Figure 9. As can be seen, the observed photochemical process is slower in the presence of chitosan, which can be represented by comparing the time needed for 75% dye degradation—in a solution of DMASQ alone it is ~500 s, while in a CS solution with 2% DMASQ—about 3500 s, i.e., seven times longer.

This process is irreversible which indicates a photochemical reaction related to the destruction of the dye’s chromophore systems. Since the UV-irradiation takes place in the air atmosphere, the most likely reaction is oxidative degradation involving the addition of oxygen to the double bond of cyclobutene with the formation of a peroxide bridge. In the next step, the unstable peroxide bond breaks, moreover, a central four-membered ring with internal stresses can also tear under the influence of the high energy of the radiation quantum. The stabilizing action of chitosan can be explained by the processes of energy transfer from the excited dye molecules to the surrounding chitosan macromolecules or its direct screening effect. 

**Figure 10:**
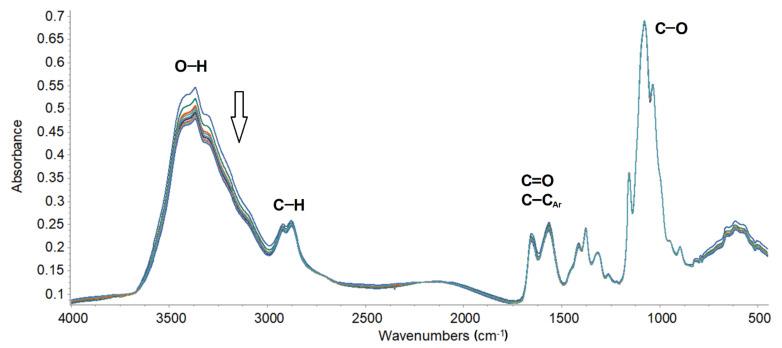
Changes in FTIR spectra of the CS film + 5% DMASQ during 6h UV irradiation (the most important bands have been assigned to appropriate groups; the arrow shows the direction of changes).

Chitosan and DMASQ doped chitosan films are resistant to UV radiation, as demonstrated by FTIR studies. Examples of ATR-FTIR spectra of a CS + 5% DMASQ film irradiated up to 6h are shown in Figure 10. During the irradiation of the samples, no significant changes in the spectra were observed, except for the decrease in the intensity of the hydroxyl band in the range of 3000–3600 cm^−1^, which is related to the gradual release of moisture adsorbed by chitosan. This process may be accompanied by the cleavage of the hydroxyl groups from the polysaccharide chains. However, the formation of new bands, e.g., carbonyl bands, was not observed, which means that there is no photooxidative degradation in the dye-doped CS film. 

The interactions between CS and dye molecules may be the reason for the good photostability of the solid-state samples. Such intermolecular interactions of a dipole nature are possible due to the presence of functional groups and short distances between them, contrary to dilute solutions, where molecules are solvated and thus separated.

Both tests in solutions and solid films indicate good system photostability, which is due to the protective effect of chitosan. The inhibition of the dye photobleaching in the presence of chitosan in solution is particularly effective.

### 3.5. Thermal Stability

**Figure 11:**
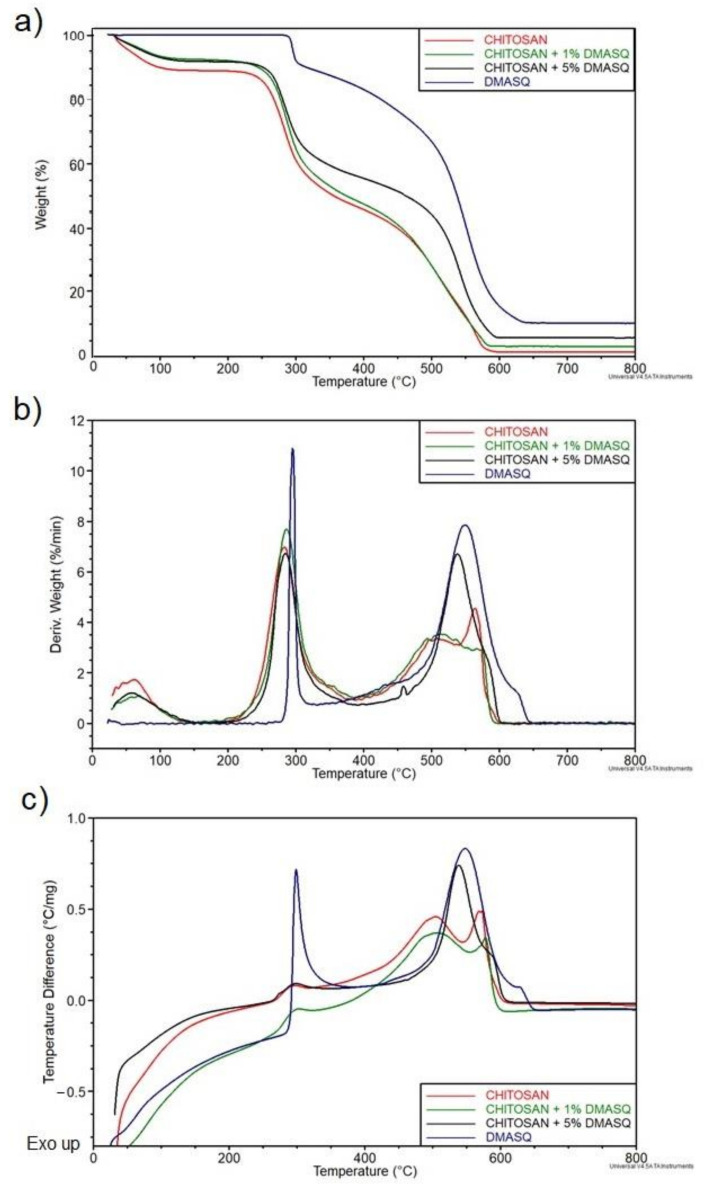
TG (**a**), DTG (**b**), and DTA (**c**) curves of chitosan, DMASQ, and chitosan with the addition of 1 and 5% dye.

The results of the dynamic thermal analysis of the tested samples are shown in Figure 11. Samples of pure and modified chitosan show the course of the TG curve typical for polysaccharides [42,43,44,45,46]. The gradual weight loss observed from the beginning of CS samples heating to about 120 °C indicates the release of trapped water molecules. It is both weakly adsorbed water and strongly bound by macromolecules capable of forming hydrogen bonds. Based on this loss, it is possible to estimate the water content in the initial samples, i.e., 11% in CS and approximately 8% in CS with 1 and 5% DMASQ. The addition of dye to CS increases T_o_ and decreases of weight loss (∆m) of the first decomposition stage indicating some improvement in the thermal stability of the CS (Table 1). Considering the second stage of decomposition of chitosan samples in the 400–600 °C range, it should be noted that the determination of T_o_ presents some difficulties since the weight loss is continuous as the temperature increases. On the other hand, the shape of peak II on the DTG and DTA curves indicates a similar course of this stage in the CS and CS with 1% DMASQ (where the peaks are double), while the CS with 5% DMASQ sample has a more similar course to the dye itself (single peak II), which is seen in Figure 11 b,c. The last two samples were also characterized by the highest degradation rate. Chitosan under oxidative conditions decomposes completely at 600 °C, while in the presence of DMASQ a carbon residue of several percent is observed.

The dye alone shows no changes in the initial stage of heating—the first conversion begins at 290 °C and reaches the maximum rate at 296 °C. It is accompanied by an 11% weight loss. This is due to DMASQ melting, as evidenced by a narrow, intense exothermic peak on the DTA curve. This confirmed the value of the melting point determined by the classical capillary tube method. Such a clear melting peak was not observed in the remaining samples (only a slight rise on the curve at about 290 °C can be noticed), which might suggest inhibition of crystallization of the dye in the CS matrix due to the interaction of macromolecules with DMASQ. However, the content of the additive is small, hence its melting peak is barely visible. The essential thermal decomposition of the dye begins after the crystalline phase melts, i.e., above 300 °C. The weight loss is continuous during further heating, but the process reaches a maximum rate of 550 °C. The carbonaceous residue after decomposition, determined at 800 °C, is ~8%. It can be a permanent cross-linked structure.

The mechanism of thermal degradation of chitosan is well known both in nitrogen and air atmosphere [42,43,44,45,46]. The main reactions, occurring in the range of 200–400 °C, are chain scission, ring-opening, and side group abstraction accompanying oxidation. Various products formed at this stage are thermally stable—they can be cross-linked structures that decompose completely above 400 °C. The main products in the end stage of thermo-oxidative degradation of CS are CO_2_, H_2_O, and a small amount of nitrogen oxides (NO_x_).

However, the effect of squaraine dye on thermal processes in CS is unknown. Like most organic compounds, DMASQ is burned under these conditions, which is confirmed by an exothermic peak on the DTA curve. It is not a complete process, as evidenced by a few percent residues after burning, present even at 800 °C. It may contain polyaromatic and strongly cross-linked structures. Taking into account the onset of decomposition (T_o_), it was found that the dye had some stabilizing effect on the CS, which could be explained by intermolecular reactions leading to the formation of covalent cross-links between DMASQ and CS molecules. Since thermal degradation takes place according to the free radical mechanism, active sites are formed both in the polymer matrix and in the dye, so their mutual recombination is possible.

### 3.6. Release of DMASQ from the Chitosan Matrix

The release of the dye from the chitosan matrix is very important from the medical point of view due to its potential application in imaging and treatment. This is of particular importance in the case of solid and semi-solid bio- and mucoadhesive formulations obtained precisely based on hydrophilic polymers like chitosan. 

For determination, if the DMASQ is stable in chitosan matrix in a liquid medium, spectrophotometric measurement of the amount of released dye in time was performed for all CS films immersed in methanol.

**Figure 12:**
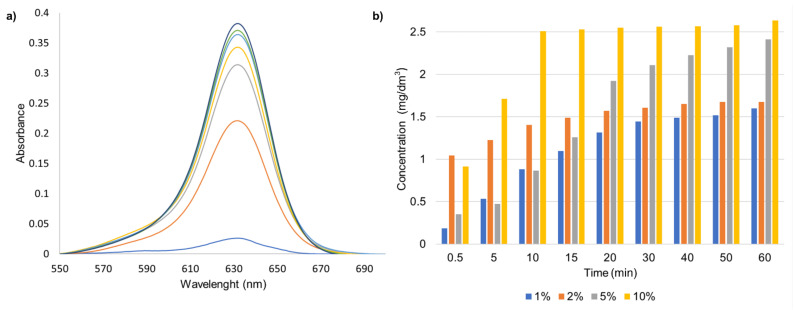
Release of the DMASQ dye from CS + 1% DMSQ chitosan film suspended in methanol–increase of absorption band in 1 h (**a**); changes of DMASQ concentration (mg/dm^3^) released in time for chitosan films with 1, 2, 5, and 10% DMASQ during 1 h, calculated based on the molar absorption coefficient ε = 75,746 M^−1^·cm^−1^ (**b**).

As can be seen in Figure 12, in all cases, regardless of the dye addition, there is little DMASQ release from the polymer matrix. The dye introduced most rapidly is re-leased in the first 15 min of immersion of the film in the solvent, while the release is still more static. Moreover, in Figure 12b it can be seen that the film containing the greatest DMASQ amount (10%) is characterized by the fastest and most dye releasing. The amount of dye released from the chitosan matrix after 60 min immersion in the solvent for all films does not exceed 2% of the DMASQ amount incorporated into the chitosan matrix, i.e., about 0.01 mg.

Due to the potential use of the obtained matrices in imaging and as possible coatings for drugs containing a fluorescent label, the obtained results are very promising. Based on the obtained dependence of the amount of released dye from time, it can be stated that these materials can be successfully used in the above-mentioned applications without losing their ability to fluorescence.

### 3.7. Potential Application of a Studied System

As mentioned before chitosan thanks to its solubility in a slightly acidic environment, biodegradability, non-toxicity, and antimicrobial activity, is a promising environmentally friendly material for the future [2,3,4,5,6,47,48,49,50]. The proposed modification of this biopolymer by chosen photosensitive compound—squaraine dye: 2,4-bis[4-(dimethylamino)phenyl]cyclobutane-1,3-diol led to obtaining a material with good photochemical and thermal resistance, both in solution and in solid-state, which suggests the possibility of various applications, among which their use in medicine and pharmacy is particularly important. In particular, chitosan solutions with small (several percent) additions of dye, showing intense fluorescence, can be used in microscopic imaging to detect infected cells in the human body. In such a system, squaraine acts as a molecular photosensor responsible for the absorption of electromagnetic radiation. On the other hand, chitosan containing functional groups (hydroxyl and amine) is capable of mucoadhesion, i.e., bioadhesion with the mucosa of living organisms—a hydrogel whose biomolecules, apart from water, contain lipids, inorganic salts, enzymes, and a glycoprotein—mucin [51]. The application and adsorption of such preparation by the diseased tissues may contribute to the gradual long-term release of the biologically active substance. 

It is known from previous literature reports that squaraine derivatives exhibit photobiological activity [48]. By contributing to the generation of reactive oxygen species (including singlet oxygen) under the influence of UV radiation, they have a destructive effect on cancer cells, hence the possibility of their use in photodynamic therapy. Stabilization of squaraine by chitosan allows for sterilization of such biomedical products.

Apart from medical applications, the use of dye-modified chitosan in the industry can be proposed [49,50]. Examples are protective coatings, sensitizers for optoelectronic or organic solar cells. In the latter case, replacement of inorganic semiconductors (now widely used in photovoltaics) with materials based on modified chitosan can be expected.

Depending on the intended applications, dye-doped chitosan should be carefully designed. In the case of a physical mixture of ingredients, the modification is relatively simple, which can make such materials widely used on a large scale.

## 4. Conclusions

The conducted studies show that chitosan stabilizes the DMASQ against high-energy UV radiation, while this dye improves the thermal resistance of CS. The solid film of DMASQ doped chitosan is a heterogeneous system but both components mix well in properly selected solvents (i.e., mixture of acetic acid and methanol, 1:1 by volume). The solutions of DMASQ doped CS allow for intensive fluorescence under 600 nm excitation, however, in solid films the emission is strongly suppressed. The advantage of this system is the strong photobleaching inhibitory effect caused by the protective action of chitosan. Moreover, the chitosan matrix also prevents rapid leaching of squaraine by methanol—as it was found, only about 2% of the dye introduced is released after an hour of soaking.

The combination of the unique properties of chitosan with the properties of squaraine dye and their mutual stabilization opens up new application possibilities for these materials.

## Figures and Tables

**Table 1:** The main parameters obtained from TG, DTG, and DTA curves of chitosan doped with DMASQ, virgin chitosan, and DMSQ dye (T_o_—onset temperature, T_max_—the temperature at maximum process rate, V_max_, ∆m—weight loss, and exo peak—position of an exothermic peak at DTA; the Roman numerals I and II denote the first and second stage of the process).

Sample	T_o_(I), (°C)	T_o_(II) (°C)	∆m(I) (%)	∆m(II) (%)	T_max_(I) (°C)/ V_max_(I) (%/min)	T_max_(II) (°C)/ V_max_(II) (%/min)	Exo Peak(I) (°C)	Exo Peak(II) (°C)
Chitosan	250	463	43.5	45.1	284/6.9	564/4.5	503	573
CS + 1% DMASQ	262	466	42.3	49.1	284/7.6	521/3.5	501	579
CS + 5% DMASQ	263	501	37.1	53.0	286/6.8	538/6.7	296	539/585*
DMASQ dye	290	498	11.3	81.2	296/15.1	550/7.9	299	549/625*

* small peak shoulder.

## Data Availability

The data presented in this study are available in Kaczmarek, H.; Rybczyński, P.; Maćczak, P.; Smolarkiewicz-Wyczachowski, A.; Ziegler-Borowska, M. Chitosan as a Protective Matrix for the Squaraine Dye.
